# Successful coronary stent implantation using directional coronary atherectomy without side branch occlusion

**DOI:** 10.1002/ccr3.6969

**Published:** 2023-02-21

**Authors:** Tomohiro Kawaguchi, Kosuke Seiyama, Masayuki Doi

**Affiliations:** ^1^ Department of Cardiology Kagawa Prefectural Central Hospital Takamatsu Japan

**Keywords:** directional coronary atherectomy, percutaneous coronary intervention, side branch occlusion

## Abstract

Percutaneous coronary intervention with a drug‐eluting stent was successfully performed without a side branch (SB) occlusion. In this case, a directional coronary atherectomy catheter played an important role in modifying the plaque at the proximal left anterior descending artery and in crossing a wire to the jeopardized SB.

## Graphical abstract

Plaque removal with a directional coronary atherectomy catheter in front of a jeopardized side branch and successful drug‐eluting stent implantation without the side branch occlusion.

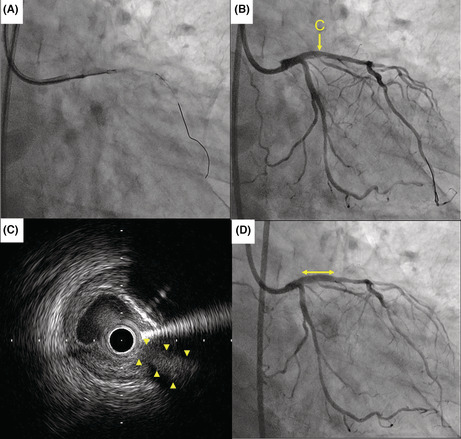

## 1 INTRODUCTION

Percutaneous coronary intervention (PCI) is an established treatment option for coronary artery disease. Formerly, a directional coronary atherectomy (DCA) catheter was favored for its effect on plaque removal and lumen enlargement.^1^, ^2^, ^3^ However, the use of this DCA catheter has decreased due to the increased frequency of coronary injury, the higher rate of periprocedural myocardial infarction, and its small contribution to improving patient prognosis.^4^, ^5^ Recently, a novel improved DCA catheter (ATHEROCUT, Nipro Corporation, Osaka, Japan) has been commercially available in Japan since 2014. Stentless PCI combined with a DCA catheter and drug‐coated balloon for proximal left anterior descending artery (LAD) lesions has been recently reported.^6^, ^7^ We report a case in which a DCA catheter was effective in plaque removal in front of the ostium of a side branch (SB) and in crossing a wire to the jeopardized SB, resulting in successful stent implantation without SB occlusion.

## 2 CASE

**FIGURE 1:**
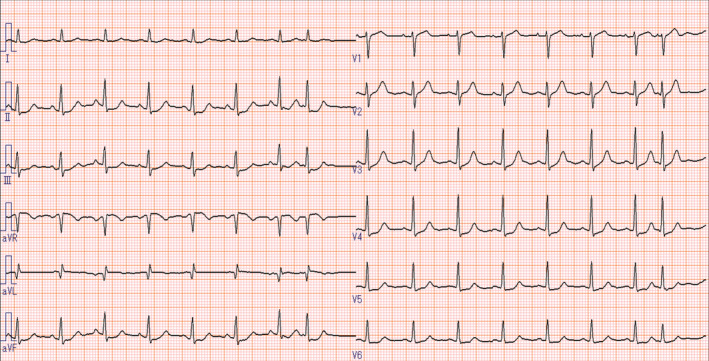
Electrocardiogram.

**FIGURE 2:**
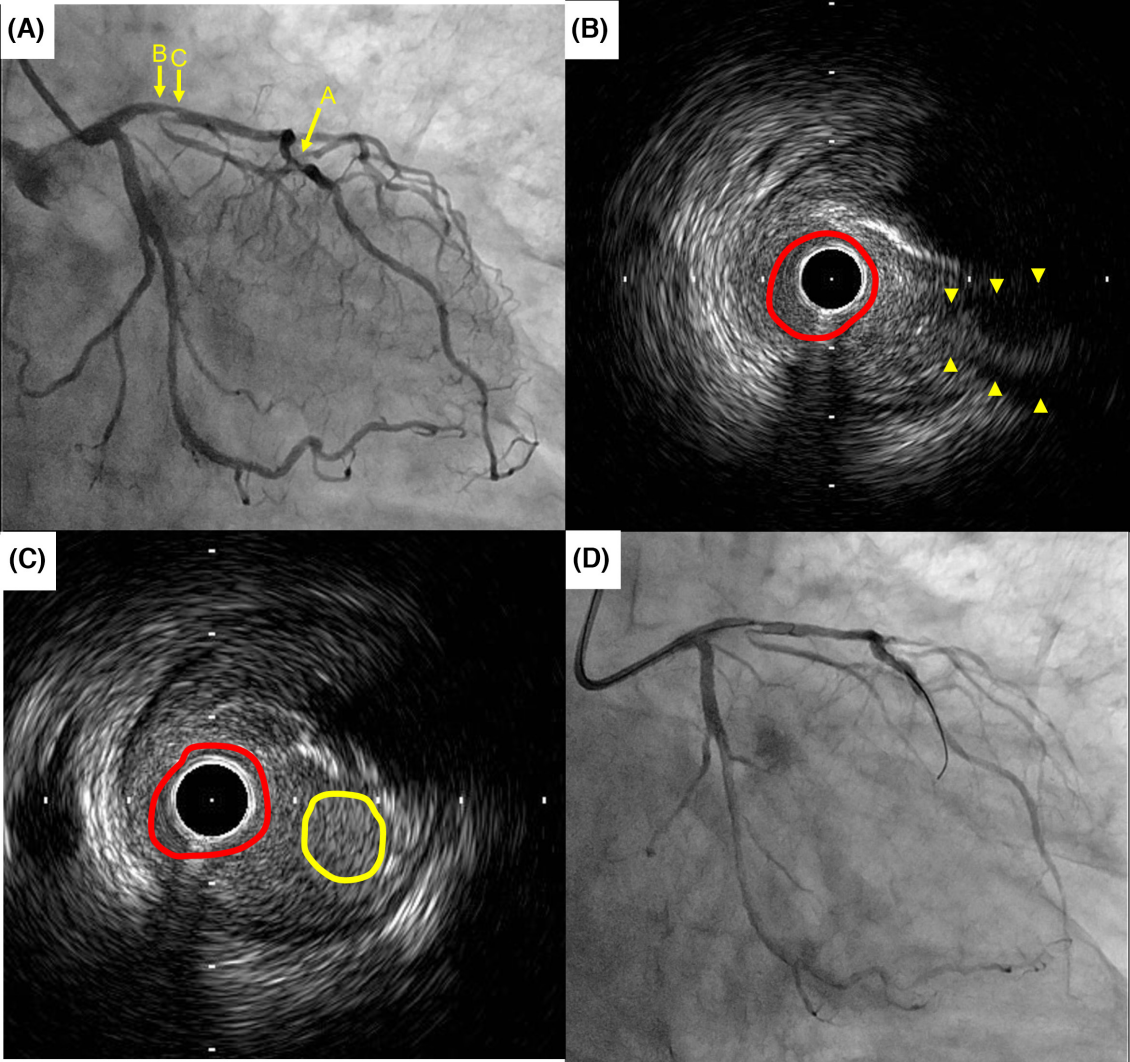
Fluoroscopic and IVUS images before atherectomy. (A) A coronary angiography. Arrow A indicates 75% stenosis at the mid‐LAD. Arrows B and C indicate the location of the IVUS images; (B) A cross‐sectional image of the ostium of the side branch (arrowheads). The circle indicates the lumen area; (C) A cross‐sectional image of the double lumen (circle). The red and yellow circles indicate the main lumen of the LAD and one leading to the side branch, respectively. (D) Failed reverse wire technique. IVUS = intravascular ultrasound, LAD = left anterior descending artery.

An 82‐year‐old man with hypertension, insulin‐dependent diabetes mellitus, and end‐stage renal disease on hemodialysis was referred to our hospital for assessment of cardiac function. He had taken 40 mg of azilsartan, 80 mg of nifedipine, 5 mg of rosuvastatin calcium, 10 mg of ezetimibe, 10 mg of raveprazole, and 28 unit of insulin, per day, respectively. Although he had no symptoms of angina, transthoracic echocardiography revealed asynergy in the anteroseptal wall of the left ventricle, and the left ventricular ejection fraction was 54% (Video S1). Electrocardiogram revealed ST depression in leads II, III, aVF, and V3‐V6 (Figure 1). Considering these results, he was deemed to be at high risk of coronary arterial disease and coronary angiography was planned. Coronary angiography revealed 75% stenosis in the proximal and mid‐LAD (Figure 2A). The fractional flow reserve value was 0.56 at the distal LAD, and an increased value was observed (0.3 at the proximal LAD and 0.1 at the mid‐LAD). Thus, he was diagnosed with silent ischemia, and these two lesions in LAD were considered as indications for revascularization via PCI. Following coronary angiography, 100 mg of aspirin and 3.75 mg of prasgrel, per day, respectively, were administrated.

**FIGURE 3:**
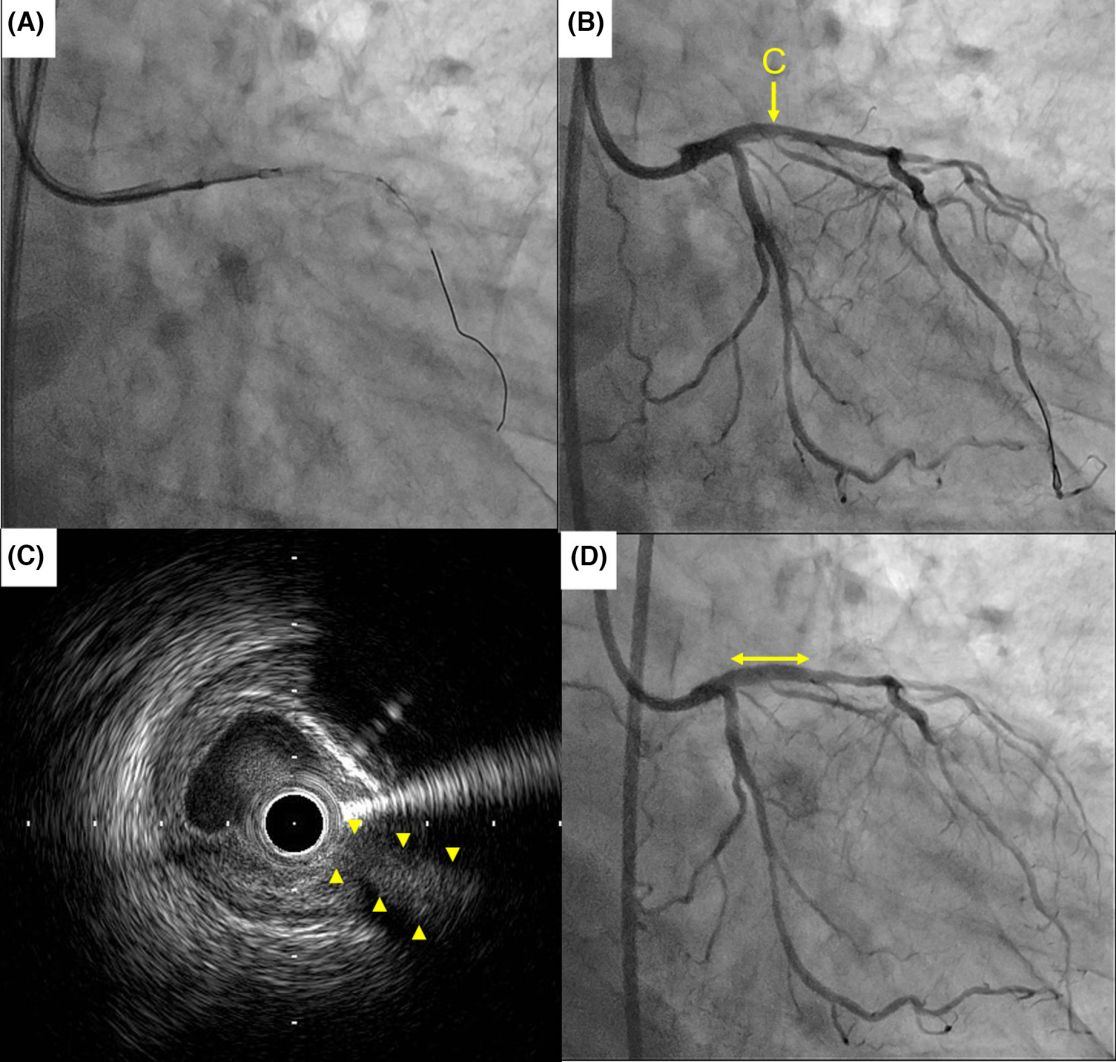
Fluoroscopic and IVUS images following atherectomy. (A) The DCA catheter; (B) Coronary angiography following atherectomy. Arrow C indicates the location of the IVUS images; (C) A cross‐sectional image of the ostium of the side branch (arrowheads); (D) The final coronary angiography. The bidirectional arrow indicates the position of the stent implantation. IVUS = intravascular ultrasound; DCA = directional coronary atherectomy.

In the first session of PCI, a 6‐Fr‐guiding catheter was inserted into the left coronary artery through the right radial artery. Thorough intravascular ultrasound (IVUS) assessment confirmed a plaque in front of the ostium of the SB, and retrograde blood flow into the SB was confirmed at the proximal LAD lesion (Figure 2B,C). Therefore, stent implantation was considered likely to occlude the branch without wire protection. A drug‐eluting stent, XIENCE Skypoint of 2.5 mm × 15 mm (Abbott Vascular, Abbott Park), was implanted into the mid‐LAD lesion. However, using the reverse wire technique (Figure 2D, Video S2), plaque penetration with a heavy tip‐loaded wire failed to cross the branch. Instead, we intended to use a DCA catheter to remove the plaque from the front of the branch, effectively allowing crossing of the wire in next session; the first session was completed. In the second session of PCI, an 8‐Fr guiding catheter was inserted via the right femoral artery, and atherectomy was then conducted with a DCA catheter (Figure 3A,B). IVUS revealed effective plaque removal and a direct connection from the LAD to the SB in the same cross‐sectional image (Figure 3C). A coronary wire was easily crossed to the SB, and drug‐eluting stent implantation with a XIENCE Skypoint of 3.0 × 15 mm was successfully performed without the SB occlusion (Figure 3D).

## 3 DISCUSSION

SB occlusion is a serious complication of PCI that leads to periprocedural myocardial damage. Procedural myocardial infarction or injury has been reported to be independently associated with cardiovascular events and long‐term mortality.^8^, ^9^, ^10^ Various techniques have been developed to avoid SB occlusion, such as the jailed balloon technique,^11^ modified jailed balloon technique,^12^, ^13^ and the jailed corsair technique.^14^ To utilize these techniques, it is necessary to cross the wire to the SB. However, technical difficulties may occasionally be encountered in challenging cases due to plaque distribution of the main branch. In our case, IVUS played an essential role in identifying the risk of SB occlusion and accessing the complex morphology of the SB. Moreover, it allows for the evaluation of the debulking effect, which is a possibility of crossing a wire to the SB. Through IVUS imaging, we speculate that the plaque location changed and covered the ostium of the SB following plaque rupture at the target lesion. In addition, the cavity created by the plaque rupture became the route of blood inflow into the branch. A treatment option considered for this lesion was the use of a cutting balloon to create antegrade blood inflow to the SB. However, this method has a possible risk of SB occlusion when antegrade inflow is not successfully created. Thus, we considered the use of a DCA catheter to be the best option for this lesion to facilitate plaque removal in front of the ostium of the SB; this approach was effective in modifying the target lesion and saving the SB in our case. Various lesions can be indications for a DCA catheter. The following lesions were reported to be suitable lesions for a DCA catheter: proximal to mid‐segment, noncalcified, large (>2.5 mm) coronary arterial segments; ostial coronary lesions; proximal LAD lesions; bifurcation stenoses; discrete saphenous vein graft stenoses; complex or thrombus‐containing lesions; highly eccentric lesions; and recurrent thrombosis or limited dissection.^15^ Ueda et al. reported that PCI in combination with IVUS‐guided DCA and a filter‐based embolic protection device was useful for prevention of distal embolization in IVUS‐derived low‐attenuated plaque, which is at high risk of distal embolization.^16^

Favorable outcomes of stentless PCI combined with a DCA catheter and drug‐coated balloon for proximal LAD lesions have been recently reported.^6^, ^7^ In our case, although stentless PCI for proximal LAD lesion was a good option, a drug‐eluting stent was implanted because the patient had already undergone stent implantation at mid‐LAD. In addition to the usefulness of the DCA catheter for stentless PCI with a drug‐coated balloon, our case showed that this approach was effective in modifying the target lesion and saving the jeopardized SB.

## 4 CONCLUSION

In this case, a directional coronary atherectomy catheter with IVUS guidance was effective and played an important role in modifying the plaque at the proximal left anterior descending artery and in crossing a wire to the jeopardized SB. With the debulking effect of a DCA catheter, PCI using a drug‐eluting stent was successfully performed without SB occlusion.

## AUTHOR CONTRIBUTIONS

TK involved in data curation and writing—original draft. KS involved in data curation. MD involved in writing—review and editing.

## FUNDING INFORMATION

None.

## CONFLICT OF INTEREST STATEMENT

None.

## INFORMED CONSENT

Written informed consent was obtained from the patient to publish this report in accordance with the journal's patient consent policy.

## Supporting information


Video S1.

Video S2.

## Data Availability

The data that support the findings of this study are available from the corresponding author upon reasonable request.
